# Quantitative assessment of temporal stability of the myocardial signal and relative blood to myocardial contrast in free breathing respiratory triggered retrospectively cardiac gated cine steady-state free precession (RT-SSFP) imaging

**DOI:** 10.1186/1532-429X-18-S1-P333

**Published:** 2016-01-27

**Authors:** Amol Pednekar, Jiming Zhang, Claudio Arena, Melissa Andrews, Debra Dees, Benjamin Cheong, Raja Muthupillai

**Affiliations:** 1 Philips Healthcare, Houston, TX USA; 2 Radiology, CHI St. Luke's Health, Houston, TX USA

## Background

The SSFP sequence must attain and maintain the magnetization steady state (SS) as any interruption will entail severe imaging artifacts. In this work: (a) we propose quantitative metrics to assess the temporal stability of the myocardial signal and the blood to myocardial contrast during a cine SSFP sequence; and (b) evaluate the performance of three cine bSSFP acquisitions using these metrics: 1) the cardiac triggered (CT) unprepared SS; 2) 1 RR SS prepared breath hold (BH); and 3) min 35 oms SS prepared respiratory triggered (RT) sequence.

## Methods

All imaging for this prospective, IRB approved study was performed on a 1.5 T commercial MR scanner (Achieva, Philips Healthcare) in 23 volunteers (10m/13f; age 44(24-60)yrs). Identical imaging parameters were used for CT, BH, and RT sequences (TR/TE/flip angle: 3/1.5/60°); acqd voxel size: 1.5-1.9 × 1.5-2.1 × 7-8 mm^3^; SENSE factor:2. Following were differences in acquisition for first 10 and subsequent 13 subjs:- temp res:30-45 ms/12-15 ms; acq time:8-10/12-15 RR intervals/slice. A time-signal intensity (SI) graph was constructed from the mean SI from regions of interest (ROI) drawn in the liver, myocardium and blood pool at mid-ventricular level. The mean Si of each ROI was normalized to the min SI across the cardiac phases, and time axis was expressed as % of the cardiac cycle. The histogram analysis was performed on the normalized intensity values. The ratio of 95 percentile to 5 percentile intensity serves as the extent of signal intensity variation (SIV). The 110% of the mode of the histogram is used is used to compute the percentage of cardiac cycle (PCC) spent in SS. The blood to myocardial contrast (BMC) was normalized to myocardial intensity.

## Results

**Figure 1:**
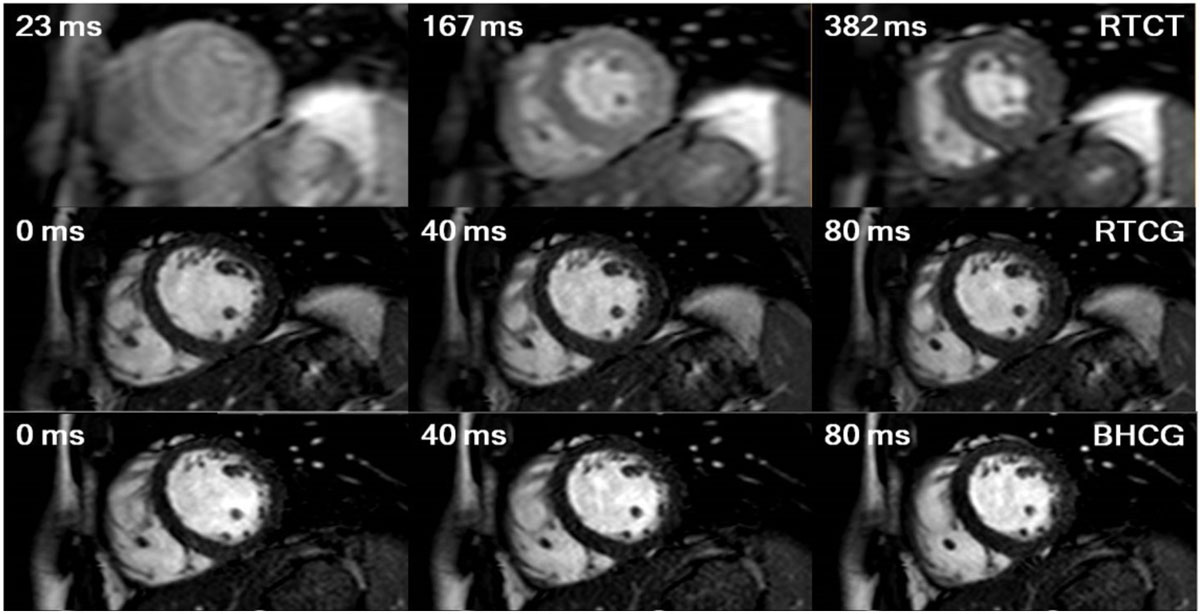
**Cine SSFP images of Subject 3 with respiratory and cardiac triggered (RTCT), respiratory trigged cardiac gated (RTCG) and breathhold cardiac gated (BHCG) acquisition techniques**. The middle row depicts the successful drive to steady state signal in 400 ms being used in the proposed RTCG segmented k-space cine SSFP acquisition.

**Figure 2:**
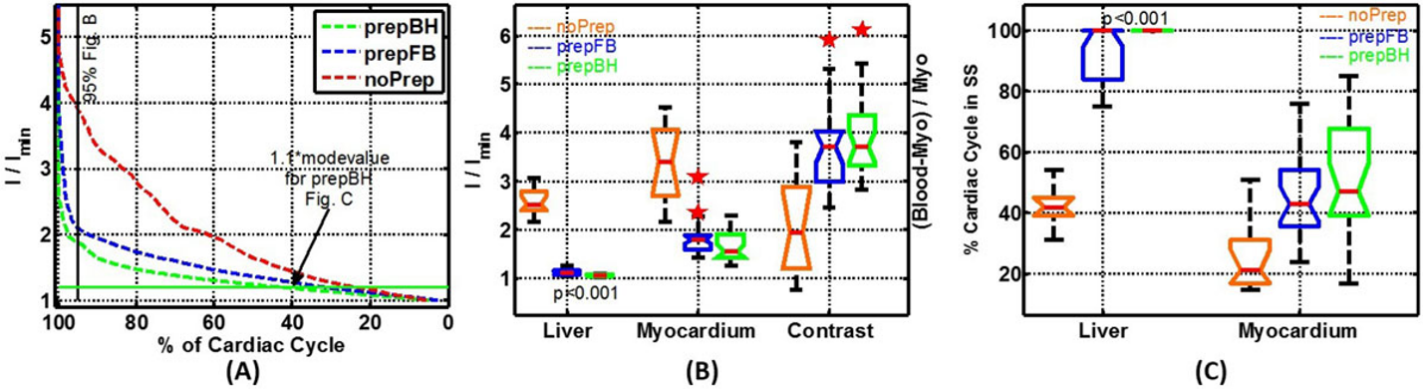
**Statistical analyses of signal intensity variation over a cardiac cycle**. (A) Cumulative density function of normalized intensity (NI) values (divided by minimum intensity for a given ROI for that subject) of myocardium (mean over all 23 subjects). (B) Distribution of ratio of 95th to 5th percentile NI for liver, myocardium and blood to myocardial contrast across subjects. (C) Percentage of cardiac cycle spent below the 1.1 times the mode value of the intensity over cardiac cycle for liver and myocardium. Plot B depicts the extent of myocardial signal variation in comparison to the contrast ration between blood and myocardium. Plot C depicts the percentage of cardiac cycle spent in steady state by the liver and myocardium.

Representative images for all 3 sequences from Subj 3 are shown in Figure 1. A cumulative distribution function of the histogram of the mean across all patients for the myocardial ROI is shown in Figure 2A. Box plots depict variation of SIV, BMC (Figure 2B) and PCC (Figure 2C) across patients. CT is statistically significantly (StS) (p < 0.001) different in all categories with respect to BH and RT. There is StS (p < 0.001) difference in SIV and PCC between liver and myocardium for BH and RT. The difference between SIV and PCC of myocardium along with BMC is insignificant for BH and RT.

## Conclusions

The starting signal is 4xSS in liver and 5xSS in myocardium and stays 3xSS and 4xSS for 95% of the cardiac cycle respectively, entailing loss of BMC along with severe flashing. The SS preparation of minimum 350 ms in RT provides equivalent SS signal as with 1 RR SS preparation in BH scans without any sacrifice in BMC. Compared to liver myocardial SIV is StS greater and PCC is Sts lower due to entrance of fresh spins into imaging plane during cardiac pulsation. The bSSFP cine cardiac is undiagnostic without the SS preparation. The minimum 350 ms SS prepared RT sequence provides myocardial SIV, PCC, and BMC equivalent to that of 1 RR SS prepared BH sequence.

